# Cerebral Venous Thrombosis with Migraine-Like Headache and the Trigeminovascular System

**DOI:** 10.1155/2016/2059749

**Published:** 2016-02-17

**Authors:** Fábio A. Nascimento, Marília Grando Sória, Vanessa Rizelio, Pedro A. Kowacs

**Affiliations:** ^1^Division of Neurology, Toronto Western Hospital, University of Toronto, Toronto, ON, Canada; ^2^Neurological Institute of Curitiba (INC), 300 Rua Jeremias Maciel Perretto, 81210-310 Curitiba, PR, Brazil

## Abstract

Cerebral venous thrombosis- (CVT-) associated headache is considered a secondary headache, commonly presenting as intracranial hypertension headache in association with seizures and/or neurological signs. However, it can occasionally mimic migraine. We report a patient presenting with a migraine-like, CVT-related headache refractory to several medications but intravenous dihydroergotamine (DHE). The response to DHE, which is considered to be an antimigraine medication, in addition to the neurovascular nature of migraine, points out to a probable similarity between CVT-headache and migraine. Based on experimental studies, we discuss this similarity and hypothesize a trigeminovascular role in the genesis of CVT-associated headache.

## 1. Introduction

Headaches attributed to cerebral venous thrombosis (CVT) (ICHD-III 6.6) are those temporally related to a diagnosis of CVT, confirmed by neuroimaging, and most commonly present as “diffuse, progressive, intense headaches, associated with other signs of intracranial hypertension” [1]. There are several other types of presentations, most of them occurring in association with focal signs (neurological deficits or seizures) and/or signs of intracranial hypertension, subacute encephalopathy, or cavernous sinus syndrome [1]. We report a patient presenting with a migraine-like CVT-related headache that was refractory to multiple medications but intravenous dihydroergotamine (DHE). Notably, the CVT involved thrombosis of the superior sagittal sinus (SSS). The response of this CVT-related headache to intravenous DHE, in addition to the fact that the headache featured migraine characteristics, highlights pathophysiological similarities between this secondary headache and migraine. Moreover, this good response resembles, in some aspects, the DHE effect in the experimental cat model whose SSS was stimulated [2].

## 2. Case Report

Eight days after bariatric surgery, a 35-year-old woman presented with left-sided motor seizures, hemiparesis, hypoesthesia, and severe headache, resembling migraine without aura. The headache was rated at 10/10 (Verbal Rating Scale), unilateral, pulsatile, associated with nausea, photophobia, and phonophobia, and aggravated by physical activity. Antecedents included rheumatic fever and pulmonary thromboembolism secondary to deep venous thrombosis. She was on enoxaparin, cefazolin, metronidazole, gentamicin, parecoxib, metamizole sodium, and nalbuphine hydrochloride. None of the analgesics, however, relieved her headache.

**Figure 1:**
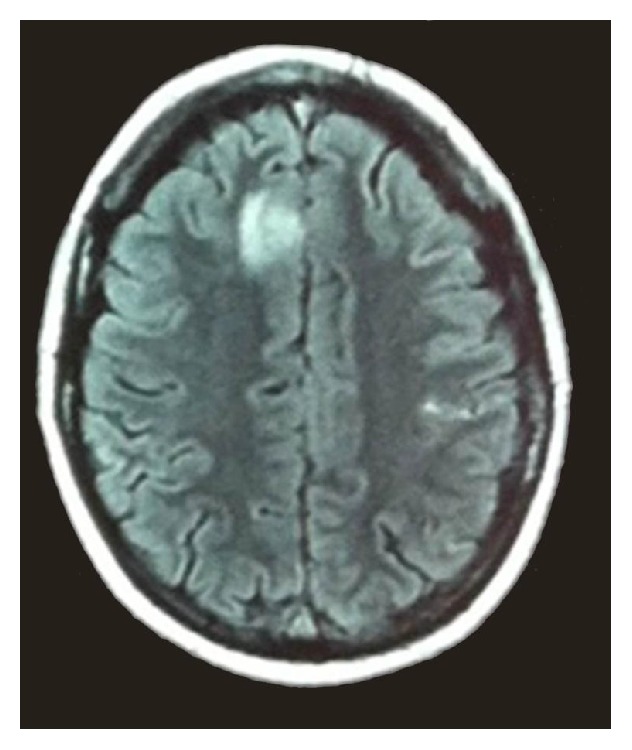
Axial FLAIR MRI shows peripheral hyperintensity in the right frontal lobe as well as abnormal high signal intensity involving the superior sagittal sinus, findings suggestive of cerebral venous thrombosis.

The patient was referred to our service, where she was investigated with a brain MRI that showed hyperintensity in the right frontal lobe and abnormal high signal intensity in the superior sagittal sinus topography, findings highly suggestive of CVT (Figure 1). We started her on oral acetazolamide 250 mg bid, warfarin sodium 5 mg od, subcutaneous enoxaparin 90 mg bid, intravenous alizapride 50 mg tid, ondansetron hydrochloride 8 mg tid, and phenytoin 100 mg tid, for the management of intracranial hypertension, SSS thrombosis, nausea, and seizures, respectively. Her migraine-like headache persisted despite intravenous metamizole sodium 1,000 mg bid, tramadol hydrochloride 100 mg qid, and morphine sulfate 0.06 mg bid. Due to the migraine features, as well as the severity and refractoriness of her headache, intravenous DHE 0.5 mg tid was administered, with subsequent resolution of her headache.

## 3. Discussion

We report a patient presenting with migraine-like headache in the context of CVT and frontal infarction. Although her headache can be readily diagnosed as being due to CVT (based on the diagnostic criteria proposed by ICHD-III, per above) [1], the possibility of the headache being secondary to the frontal infarction should be acknowledged. The latter hypothesis, nevertheless, is unlikely, mainly because our patient's headache did not have a self-limited course (which is characteristic of headache attributed to cerebral infarction) and because her headache is better accounted for by another ICDH-III diagnosis (6.6, headache attributed to cerebral venous thrombosis) [1]. Based on the fact that our patient's headache resolved after the use of intravenous DHE, we believe that there is a link between this migraine-like type of CVT-related headache and migraine. One may state that the DHE relieved the patient's headache because it was in fact secondary to intracranial hypertension (ICH). However, we believe this hypothesis is highly unlikely because (1) the patient's headache had features of migraine, as it was severe in intensity, pulsatile, unilateral, associated with nausea/photophobia/phonophobia, and aggravated by exertion and lacked similarities with ICH headache; and (2) although DHE has been anecdotally reported to control ICH, this finding was not further confirmed [3]. Hence, intravenous DHE remains an effective option for treating refractory migraine headaches in emergency settings [4].

### 3.1. Parallelism with Experimental Models

**Figure 2:**
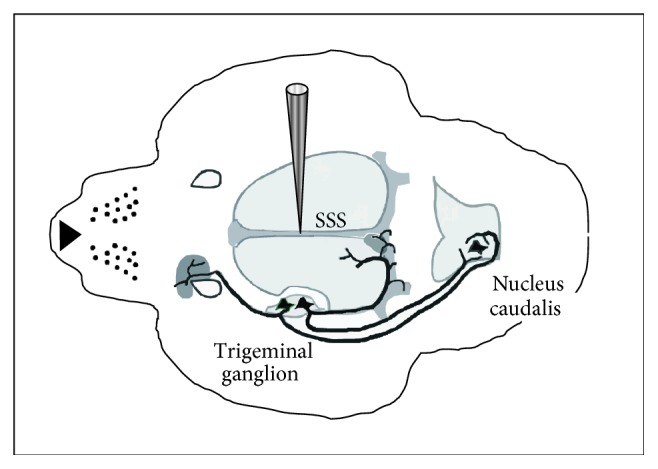
Sensitization of central trigeminovascular circuitry by stimulation of the superior sagittal sinus (SSS). Figure modified from [11].

There is a parallel between our patient's CVT-associated headache (due to thrombosis of the SSS), which had migraine features and responded with DHE administration, and the experiment involving a cat as a migraine surrogate whose SSS was stimulated [2].

(Figure 2) Our report is essentially the reproduction “in anima nobilis” of the SSS model. The trigeminovascular system (TVS) plays an essential role in the pathophysiology of migraine and is supposedly the “peripheral arm” of migraine pathways [5, 6]. The TVS is the nociceptive input from the large cerebral vessels, the large venous sinuses (such as the SSS), and the dura mater, while the thalamus and the cortex are responsible for pain-related sensations [6]. Indeed, the TVS seem to account for migraineurs' headache and for extracranial allodynia [7]. Detection of increased levels of calcitonin gene-related peptide (CGRP) in the blood collected from the jugular vein of migraineurs during migraine attacks not only confirmed the neurovascular nature of migraine but also established a correlation with the contemporary animal model, as mentioned above [2].

### 3.2. Nosology of CVT-Related Headache

The ICHD-III states “if a new headache occurs for the first time in close temporal relation to another disorder that is known to cause headache, or fulfils other criteria for causation by that disorder, the new headache is coded as a secondary headache attributed to the causative disorder.” While migraine is considered to be a neurovascular headache, CVT-associated headaches are considered to be related to increased intracranial pressure. However, it has been hypothesized that sinus wall changes could play a role in cases without intracranial hypertension [8]. It is important to recognize that headache disorders caused by CVT may present with symptoms belonging to many conditions, including migraine disorders [1, 8, 9]. It has also been reported that transverse sinus stenosis was frequent in patients with idiopathic intracranial hypertension without papilledema presenting as primary headaches, as chronic tension type headaches and migraine headaches. However, neither were their cases acute nor was there mention to response to DHE. Although that headache is recognized as the most frequent symptom in CVT, its mechanisms remain unknown [8].

### 3.3. Dihydroergotamine Effects in Migraine Headaches and in the TVS

Dihydroergotamine, which is effective in resolving migraine, also proved to inhibit the central activation of the trigeminovascular pathway in the cat model [5], as other antimigraine drugs [2].

DHE antimigraine efficacy relates to blockage of neurogenic inflammation in meningeal tissues and nociceptive transmission in the trigeminal nucleus caudalis. Their agonist action on 5-HT_1B_, 5-HT_1D_ and perhaps on 5-HT_1F_ receptors provokes trigeminal inhibition and meningeal vasoconstriction. DHE is also a venoconstrictor due to its action on alpha-adrenoceptors [6]. Nevertheless, vasoconstriction properties are more related to ergotamine than to DHE. Although there has been a study arguing against the use of DHE in patients with coronary, cerebral, or peripheral vascular disease [10], we failed to find any other report associating adverse cerebrovascular outcomes with DHE usage.

### 3.4. Inferences from This Case

The interruption of the migraine-like headache in the above-mentioned patient after intravenous DHE leads us to hypothesize that, in this case, CVT played a role akin to the SSS model. The placement of this condition under an “aetiologic umbrella” may not only elude the correct interpretation of the nature of the pain, but also interfere with an effective therapeutical approach.

Dosing CGRP in blood collected from the jugular vein of treatment-naive patients with suspected CVT would confirm or reject this hypothesis. If confirmed, migraine-like headache related to CVT or to other medical “neurovascular” conditions should perhaps be classified according to the underlying pathophysiology. However, these conditions (if classified in terms of primary versus secondary) should still be considered as secondary headaches despite sharing characteristics of a primary headache, in this case, migraine. Moreover, this case supports the idea that migraine attacks are a common reaction pattern, which can be deflagrated not only by the brainstem “migraine generator” but also by noxious stimulation of other components pertaining to the trigeminovascular system.

## Abbreviations

ICHD: International Classification of Headache Disorders
CVT: Cerebral venous thrombosis
DHE: Dihydroergotamine
SSS: Superior sagittal sinus
TVS: Trigeminovascular system
CGRP: Calcitonin gene-related peptide.

## References

[1] Headache Classification Committee of the International Headache Society (HIS) (2013). The international classification of headache disorders, 3rd edition (beta version). *Cephalalgia*.

[2] Goadsby P. J., Edvinsson L. (1993). The trigeminovascular system and migraine: studies characterizing cerebrovascular and neuropeptide changes seen in humans and cats. *Annals of Neurology*.

[3] Bundgaard H., von Oettingen G., Jørgensen H. A., Jensen K., Cold G. E. (2001). Effects of dihydroergotamine on intracranial pressure, cerebral blood flow, and cerebral metabolism in patients undergoing craniotomy for brain tumors. *Journal of Neurosurgical Anesthesiology*.

[4] Klapper J. A., Stanton J. (1993). Current emergency treatment of severe migraine headaches. *Headache*.

[5] Hoskin K. L., Kaube H., Goadsby P. J. (1996). Central activation of the trigeminovascular pathway in the cat is inhibited by dihydroergotamine. A c-Fos and electrophysiological study. *Brain*.

[6] Goadsby P. J. (2005). Migraine pathophysiology. *Headache*.

[7] Cumberbatch M. J., Williamson D. J., Mason G. S., Hill R. G., Hargreaves R. J. (1999). Dural vasodilation causes a sensitization of rat caudal trigeminal neurones in vivo that is blocked by a 5-HT(1B/1D) agonist. *British Journal of Pharmacology*.

[8] Cumurciuc R., Crassard I., Sarov M., Valade D., Bousser M. G. (2005). Headache as the only neurological sign of cerebral venous thrombosis: a series of 17 cases. *Journal of Neurology, Neurosurgery and Psychiatry*.

[9] Agostoni E. (2004). Headache in cerebral venous thrombosis. *Neurological Sciences*.

[10] Saper J. R., Silberstein S. (2006). Pharmacology of dihydroergotamine and evidence for efficacy and safety in migraine. *Headache*.

[11] Bernstein C., Burstein R. (2012). Sensitization of the trigeminovascular pathway: perspective and implications to migraine pathophysiology. *Journal of Clinical Neurology*.

